# Legal Notes for Institutional Workers

**Published:** 1915-05-01

**Authors:** 


### LEGAL NOTES FOR INSTITUTIONAL WORKERS.

### The Insurance Commissioners' Regulations Challenged.

As is well known, the scheme of the Insurance Act was that the details should be worked out by regulations made by the Commissioners. The statute also provides that every Insurance Committee shall "make provision for the proper and sufficient supply of drugs and medicines and prescribed appliances in accordance with regulations made by the Insurance Commissioners." Provision had been so made by the various committees, but there seemed to be a feeling in some quarters, shared by the Insurance Commissioners, that there was on the part of some panel doctors a tendency to over-prescribe, and a want of effective control on the part of some Insurance Committees. The panel druggists were stated to have been affected in their net remuneration by excessive prescribing, and difficulties in continuing agreements with them were threatened. The Scottish Insurance Commissioners proposed to institute a process of uniform and comprehensive audit, and prepared and published regulations thereanent; and these they were in terms of the statute on the point of laying before Parliament, when a process of interdict was raised by the Insurance Committee of the Burgh of Glasgow on the ground that the proposed regulations were *ultra vires* of the Commissioners and an invasion of the statutory functions and right of independent action of the Committee. The Court held that they had no jurisdiction to entertain any question as to the validity of the regulations, and dismissed the interdict. The regulations made by the Commissioners were declared, in the Insurance Act itself, to be of the same effect as if they were contained in the Act. How then, it was pointed out, could they be open to review and consideration by the Courts at all? The Courts have no more to do with regulations so made than they have with Acts of Parliament. If alteration is desired, the remedy must accordingly be sought by a change in the law, not by an appeal to the Courts.

### Capacity for Light Work.

Another difficult question under the Workmen's Compensation Act has again been before the Courts—namely, the capacity of an injured workman for "light work," and the consequent reduction of the compensation which the employer will have to pay. The earlier cases on the Act lay down that the employer ought to show what particular light work the injured workman is capable of performing, and that he can obtain it in his maimed condition; but now the Court of Appeal have taken a different line, and held that a workman is not entitled to stand by and make no attempt whatever to procure employment, for to rest content to live as a pensioner on the weekly payment that is allowed to him by his employers is not what the statute contemplated. If, therefore, the Court are satisfied of the practicability of the workman, if he sincerely wishes it, obtaining something to do, they can reduce the compensation to such sum as they think proper. Here, again, the judge trying such a question must be largely indebted to skilled evidence, some of which will certainly be of a medical nature, and some, we suggest, might be contributed by the directors and officials of the local labour exchanges, who could best and most impartially inform the Court of the state of the labour market in the district in which the workman lived.

